# Seroprevalence of Q fever, Brucellosis, and Bluetongue in Selected Provinces in Lao People's Democratic Republic

**DOI:** 10.4269/ajtmh.15-0913

**Published:** 2016-09-07

**Authors:** Bounlom Douangngeun, Watthana Theppangna, Vilayvahn Soukvilay, Chanthana Senaphanh, Kamphok Phithacthep, Souk Phomhaksa, Samuel Yingst, Eric Lombardini, Eric Hansson, Paul W. Selleck, Stuart D. Blacksell

**Affiliations:** ^1^ National Animal Health Laboratory, Department of Livestock and Fisheries, Ministry of Agriculture and Forestry, Vientiane, Lao People's Democratic Republic; ^2^ Armed Forces Research Institute of Medical Sciences, Bangkok, Thailand; ^3^ Mahidol-Oxford Tropical Medicine Research Unit, Faculty of Tropical Medicine, Mahidol University, Bangkok, Thailand; ^4^ Centre for Tropical Medicine, Nuffield Department of Clinical Medicine, Churchill Hospital, Oxford, United Kingdom; ^5^ Microbiology Laboratory, Lao-Oxford-Mahosot Hospital-Wellcome Trust Research Unit, Mahosot Hospital, Vientiane, Lao People's Democratic Republic

## Abstract

This study has determined the proportional seropositivity of two zoonotic diseases, Q fever and brucellosis, and bluetongue virus (BTV) which is nonzoonotic, in five provinces of Lao People's Democratic Republic (PDR) (Loungphabang, Luangnumtha, Xayaboury, Xiengkhouang, and Champasak, and Vientiane Province and Vientiane capital). A total of 1,089 samples from buffalo, cattle, pigs, and goats were tested, with seropositivity of BTV (96.7%), Q fever (1.2%), and brucellosis (0.3%). The results of this survey indicated that Q fever seropositivity is not widely distributed in Lao PDR; however, Xayaboury Province had a cluster of seropositive cattle in seven villages in four districts (Botan, Kenthao, Paklaiy, and Phiang) that share a border with Thailand. Further studies are required to determine if Xayaboury Province is indeed an epidemiological hot spot of Q fever activity. There is an urgent need to determine the levels of economic loss and human health-related issues caused by Q fever, brucellosis, and BTV in Lao PDR.

Lao People's Democratic Republic (Lao PDR) is a largely agricultural society with a reliance on livestock farming to sustain livelihoods.^1^ Furthermore, farmers and those involved in the raising of animals have a close working relationship with their animals and, in the case of zoonotic diseases, have the potential for transmission between humans and livestock.^1^,^2^ This study determined the antibody seropositivity of three diseases in five provinces of Lao PDR; two zoonoses: Q fever (causative agent *Coxiella burnetii*) and brucellosis, and bluetongue virus (BTV) which is nonzoonotic but has the potential to cause significant negative economic impacts.

A total of 1,089 serum samples were collected from buffalo, cattle, pigs, and goats from the four northern provinces of Loungphabang, Luangnumtha, Xayaboury, and Xiengkhouang, the southern province of Champasak, and Vientiane Province and Vientiane capital in the central area (Figure 1

**Figure 1.**
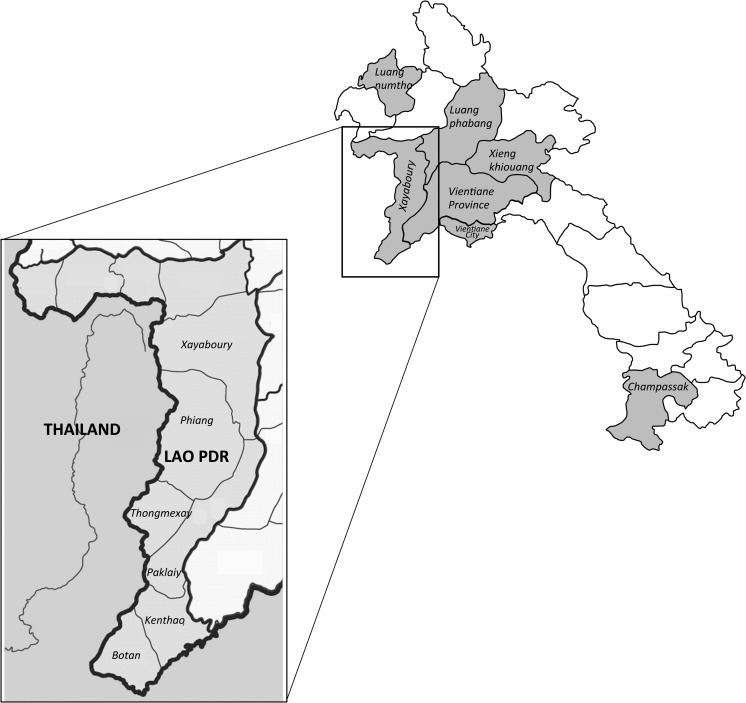
Provinces where samples were collected in Lao People's Democratic Republic. Inset details districts within Xayaboury Province that share a border with Thailand.

). A total of 16 districts and 58 villages within the selected provinces were sampled. The samples were collected as part of foot and mouth disease postvaccination surveillance activities during the period September 2013–July 2015. The enzyme-linked immunosorbent assay (ELISA) for the detection of BTV antibodies was a competitive ELISA (ID-Vet, Gabrels, France) similar to the one previously described,^3^ whereas the tests for the detection of Q fever (ID-Vet) and brucellosis (ID-Vet) antibodies were semiquantitative indirect ELISAs. The Rose Bengal agglutination test was used to corroborate the result of brucellosis antibody–positive ELISA tests. Provincial and district seroprevalence results were examined for statistical significance (*P* < 0.05) using χ^2^ (for contingency table frequencies > 5) and Fisher's exact (frequencies ≤ 5) tests.

Results indicated a high proportion of BTV antibodies in Lao PDR. Six hundred and sixty two cattle, buffalo and goat sera were tested for antibodies against BTV by the ID-Vet competitive ELISA kit. In the five provinces from which buffalo and cattle were sampled, 96.7% of the animals demonstrated antibodies against BTV, and there was significant difference between seroprevalences in different provinces (Fisher's exact, *P* = 0.003), although this observation is likely to be of no biological or epidemiological relevance due to the high BTV seroprevalence. All but 22 sera were positive for BTV antibody according to the cutoff criteria of the kit. All six goat sera tested were positive. Pigs are not normally associated with BTV infection and therefore were not tested in the study. Six hundred and sixty two cattle, buffalo, and goat sera were tested for antibodies against BTV by the ID-Vet competitive ELISA kit. The 22 negative sera were from both cattle and buffalo. Bluetongue is transmitted by multiple species of biting *Culicoides* sp. midges and is primarily a clinical disease of sheep and goats resulting in acute disease often leading to death^4^; however, there have been no reported cases of clinical bluetongue in Lao PDR. Cattle and buffalo may act as asymptomatic amplifying hosts,^4^ and may also have reproductive consequences including infertility, abortion, fetal mummification, stillbirths, and congenital anomalies and dysfunctions in the live offspring.^5^,^6^ Bluetongue was recently introduced into Europe where it caused significant economic losses,^7^ and there is also need to determine the economic consequences of BTV infections in Lao PDR resulting from reproductive losses. There are 26 serotypes of virus that cause disease worldwide,^4^ but there is limited contemporary information regarding the epidemiology of BTV in Asia. Epidemiological studies based on sentinel herds in Indonesia and Malaysia have isolated BTV serotypes 1, 2, 3, 7, 9, 12, 16, 21, and 23.^8^,^9^ China has reported BTV serotypes 1, 2, 3, 4, 9, 11, 12, 15, 16, 21, and 23 in the mid-late 1990s in Yunnan Province,^10^,^11^ which shares a common border with the northern provinces of Lao PDR including Luangnumtha province. BTV serotype 21 has been reported in Australia, India, Indonesia, China, and Japan,^12^ and BTV serotypes 2 and 12 have been reported in Taiwan.^13^ There is no BTV epidemiological information from Thailand, Cambodia, Vietnam, and Myanmar. There remains a need to determine what BTV serotypes are circulating in Lao PDR and neighboring countries.

The results of this survey indicated that Q fever antibodies are not widely distributed in Lao PDR, with only 13 (1.2% overall) antibody-positive cattle samples located in Xayaboury (9; 3.7%), Luangnumtha (3; 1.8%), and Xiengkhouang (1; 1.6%) provinces (Tables 1 and 2) with significant difference between the provincial seropositivity results (Fisher's exact *P* = 0.001). All Q fever antibody–positive animals were cattle (χ^2^ 14.08, *P* = 0.003). Interestingly, Xayaboury Province had a clustering of seropositive cattle in seven villages in four districts (Botan, Kenthao, Paklaiy, and Phiang) (Table 3) that share a border with Thailand (Figure 1), where Q fever is endemic.^14^–^18^ This may be an epidemiologic significant observation with two of the seropositive villages (Nasarn 33.2%; 2/6 positive and Jomphet 50%; 2/4 positive) having two positive animals in the village (Table 3). In the previous study of Vongxay and others,^19^ the overall seropositivity was similar (i.e., 4.0%) and Xayaboury Province had a seropositivity rate of 15.9%. Further studies including possible shedding of *Coxiella burnetii* by using polymerase chain reaction on vaginal swabs and milk in farm animals are required to determine whether Xayaboury Province is indeed an epidemiological hot spot of Q fever activity in Lao PDR.

The results presented from this survey indicated that brucellosis antibodies are not widely distributed throughout Lao PDR with an overall seropositivity rate of 0.3% with no significant difference between the provincial seropositivity results (Fisher's exact *P* = 0.471). All three positive cattle sera were tested using the Rose Bengal agglutination test to corroborate the result. The previous study of Vongxay and others^19^ gave a similar result (i.e., 0.2%). Five sera that were negative according to the cutoff criteria of the ID-Vet ELISA kit that had results close to the cutoff were also tested in the Rose Bengal test, with three of these giving weak positive reactions. This suggests that the cutoff of the ID-Vet ELISA kit is set high to maximize the diagnostic specificity and avoid false positive results; however, it is recognized that a limitation of this study is the need to confirm brucellosis antibody results using compliment fixation test, but this was not available at the time of testing.

The main limitations of this study were that sample numbers were limited to just over 1,000; not all species were sampled from all provinces and the small number of goats sampled. It would be optimal to test a larger sample size of all species from all provinces to obtain greater statistical power and this was emphasized with statistical difference noted between the species for Q fever but not the other diseases (Q fever *P* = 0.003, BTV *P* = 0.603; brucellosis *P* = 0.366). Also, it is possible that seropositivity is underestimated due to the use of serological tests that have a low sensitivity as they are designed as screening tests for disease in a western country setting. In the context in which we used the tests, we were testing for the presence of antibodies rather than the presence of disease and so the determination of an antibody cutoff in a particular geographical location may be influenced by a number of factors and may also influence the accuracy of the test especially regarding false negatives.

Results presented here indicate that BTV and Q fever are epidemiologically important, and additional studies are required to determine the true spatial distribution of the disease in Lao PDR to determine the zoonotic and disease impact in a community context.

## Figures and Tables

**Table 1:** Distribution of species tested from the various provinces in Lao People's Democratic Republic

Province	Districts	Villages	Buffalo	Cattle	Goats	Pigs	Total
Champasak	4	4	0	0	0	243	243
Loungphabang	1	2	27	0	0	0	27
Luangnumtha	1	1	0	161	0	0	161
Vientiane City	2	3	0	0	0	184	184
Vientiane Province	2	5	6	163	0	0	169
Xayaboury	5	43	66	172	6	0	244
Xiengkhouang	1	1	31	30	0	0	61
Total	16	59	130	526	6	427	1,089

**Table 2:** Proportional seropositivity of bluetongue, Q fever, and brucellosis antibodies in Lao People's Democratic Republic

Province	Total	BTV	Q fever	Brucellosis
Champasak	243	NT	0	0
Loungphabang	27	27 (100%)	0	0
Luangnumtha	161	158 (98.1%)	3 (1.8%)	1 (0.1%)
Vientiane City	184	NT	0	0
Vientiane Province	169	155 (96.3%)	0	0
Xayaboury	244	241 (98.8%)	9 (3.7%)	2 (0.2%)
Xiengkhouang	61	59 (96.7%)	1 (1.6%)	0
Total	1,089	640 (96.7%)	13 (1.2%)	3 (0.3%)
Species	Total	BTV	Q fever	Brucellosis
Buffalo	130	124 (95.4%)	0	0
Cattle	526	510 (97.0%)	13 (2.5%)	3 (0.6%)
Goats	6	6 (100%)	0	0
Pigs	427	NT	0	0
Total	1,089	640 (96.7%)	13 (1.2%)	3 (0.3%)

BTV = bluetongue virus, NT = not tested.

**Table 3:** Distribution of Q fever seropositivity in the districts of Xayaboury Province

District	Villages	Samples (*n*)	Q fever positive	Positivity per village
Botan	6	36	3 (8.3%)	Nasarn (33.2%; 2/6), Taling (16.6%; 1/6)
Kenthao	8	43	2 (4.2%)	Dongsamsan (16.6%; 1/6), Thaoken (16.6%; 1/6)
Paklaiy	11	59	2 (3.4%)	Kengsao (16.6%; 1/6), Nasak (16.6%; 1/6)
Phiang	10	58	2 (3.4%)	Jomphet (50%; 2/4)
Thongmexay	7	42	0	
Xayaboury	1	6	0	
Total	43	244	9 (3.7%)	
